# Feasibility Study for a Randomised Controlled Trial for the Topical Treatment of Impetigo in Australian General Practice

**DOI:** 10.3390/tropicalmed6040197

**Published:** 2021-11-09

**Authors:** Hilary Gorges, Leanne Hall, Clare Heal

**Affiliations:** College of Medicine and Dentistry, James Cook University, Mackay, QLD 4740, Australia; hilary.gorges@health.Qld.gov.au (H.G.); leanne.hall@jcu.edu.au (L.H.)

**Keywords:** antiseptics, antibiotic stewardship, skin infection

## Abstract

Impetigo affects millions of children worldwide. Most guidelines recommend antibiotics as first-line treatment; however, topical antiseptics present a potentially valuable, understudied, antibiotic-sparing treatment for mild impetigo. We aimed to determine the feasibility of a randomised controlled trial (RCT) comparing efficacy of soft white paraffin (SWP), hydrogen peroxide (H_2_O_2_) and mupirocin for mild impetigo. Participants were recruited from general practices and randomly assigned one of three treatments. Size and number of lesions were measured at the initial consultation and day six. Post-recruitment, interviews with general practitioners were transcribed and themes identified to determine protocol acceptability, recruitment barriers and avenues to improve delivery. Two participants received SWP (*n* = 1) and mupirocin (*n* = 1). Both commenced oral antibiotics following failure of assigned topical treatment in which lesions increased in size or number. Recruitment barriers included reduced presentation of impetigo due to COVID-19, pre-treatment with existing at-home medications and moderate/severe infection. Childcare centers and pharmacies were identified as alternative venues to improve the recruitment rate. Valuable insight was gained into the practicality of conducting a RCT of impetigo treatments in general practice. Future trials should consider recruiting outside of general practice clinics to capture patients at earlier, more mild stages of infection. Further investigation into the prevalence and impact of use of at-home expired antibiotics may be beneficial.

## 1. Introduction

Impetigo is a common childhood bacterial skin infection usually caused by *Staphylococcus aureus* or *Streptococcus pyogenes* [1,2]. The disease burden is greatest in lower socioeconomic countries and in communities struggling with overcrowding and poorer resources [2]. At any one time, 162 million children worldwide are affected by impetigo, with the highest prevalence in remote Australian First Nations’ populations [2]. It is generally a mild condition, which may be self-limiting [1]; however, in remote Australian communities where impetigo is usually caused by *S. pyogenes* [3], long-term morbidity related to post-streptococcal complications is a concern [4].

Antimicrobial resistance is a serious threat to global public health [5,6], compromising our ability to treat bacterial infections. In recent years, antimicrobial stewardship research has focused on upper respiratory tract infections, while impetigo remains an understudied area of antibiotic misuse. Resistance has already developed to some of the most common antibiotics used to treat impetigo including mupirocin [7] and fusidic acid [8]. Further overuse and misuse of antibiotics in treating impetigo may accelerate the emergence of resistant organisms [5].

In Australia, antibiotics are the mainstay of impetigo treatment, with topical antibiotics recommended for mild infections and oral antibiotics reserved for severe cases [9]. First Nations’ populations are an exception as oral antibiotics are recommended for all cases due to the risk of post-streptococcal complications [2,10]. Topical treatment is consistently recommended for mild cases worldwide; however, the type of topical treatment differs by country [11]. The evidence for many impetigo treatment guidelines, including the Infectious Diseases Society of America (IDSA) [12] and the NICE guidelines in the United Kingdom (UK) [13], is based on a 2012 Cochrane review [14]. While the efficacy of antibiotics to treat impetigo is unquestioned, the usefulness of antiseptics is unclear. The Cochrane review showed marginal superiority of topical antibiotics compared with topical antiseptics; however, this was based on two low-quality, older studies [15,16]. As such, this review concluded that there was insufficient evidence on the use of topical antiseptics [14]. The quality of this evidence creates uncertainty, which is reflected in inconsistencies between treatment guidelines worldwide. Many countries recommend the use of antiseptics as first-line treatment rather than antibiotics, while others discourage antiseptic use or make no reference to them at all [11]. These discrepancies and the rising rates of resistance to impetigo treatments highlight the need for further investigation into the efficacy of topical antiseptics for the treatment of impetigo.

Due to the paucity of evidence for the efficacy of antiseptics, current treatment recommendations are either overlooking a potentially valuable antibiotic-sparing treatment or recommending the use of an ineffective treatment. Given the importance of antimicrobial stewardship and the potential value of topical antiseptics as an antibiotic-sparing treatment for impetigo, further evidence is required to clarify their effectiveness to treat impetigo.

This study aimed to determine the feasibility of a randomised controlled trial (RCT) comparing the efficacy of soft white paraffin (SWP), topical hydrogen peroxide (H_2_O_2_) and topical mupirocin in the treatment of patients with mild impetigo.

## 2. Materials and Methods

### 2.1. Study Design

We conducted a feasibility study for a prospective RCT. This study was designed in keeping with the SPIRIT statement and reported in accordance with the CONSORT statement for pilot RCTs. The protocol was designed to compare treatment with mupirocin to H_2_O_2_ and SWP ointments for patients presenting with mild impetigo. This study was approved by the James Cook University Human Research Ethics Committee (HR7955) and registered with the Australian New Zealand Clinical Trials Registry (ACTRN12619001366145p).

### 2.2. Setting

This study was conducted in two private general practices in Mackay, Queensland, Australia. Mackay is a rural center in tropical North Queensland with a population of 131,640 [17]. Mackay is 400 km from the nearest tertiary center and 1000 km from the nearest capital city, Brisbane.

Two GP practices were purposely selected for recruitment based on previous successful participation in skin related RCTs [18,19]. All GPs who worked at both practices were recruited and given a GP information sheet before providing written informed consent. Patient recruitment was scheduled to begin in March 2020 but was delayed due to the COVID-19 pandemic until 27 October 2020 and continued until 31 January 2021. All eligible participants (or guardians) were given a participant information sheet and provided written informed consent prior to admission to this study.

To assess protocol acceptability, feasibility of recruitment and provide insight into treatment efficacy, we aimed to recruit 30 patients, 10 for each treatment arm, with an additional 3 patients to counter potential attrition.

### 2.3. Outcomes

To determine the feasibility of a larger RCT, formal semi-structured group interviews were conducted with participating GPs by the primary author at the participating practices within one week of the end of recruitment. GPs were asked to give feedback regarding (a) barriers to recruitment (b) improvements on trial delivery and (c) acceptability of protocol. The interviews were transcribed, and the content hand coded to identify major themes relating to the three key questions. Participant feedback of the treatment regime was collected by a free text feedback section of the treatment diary. Results were analysed descriptively. As this was a feasibility study, we did not conduct statistical analysis to compare outcomes between interventions.

### 2.4. Participants

Consecutive patients over the age of 2 years that presented with mild impetigo (3 lesions or less) were invited to participate. Participants were excluded if they had systemic infection (e.g., fever, nausea, and vomiting); lesions not able to be covered by dressings (e.g., near the nose or mouth); allergies to mupirocin, SWP or H_2_O_2_; currently on antibiotic therapy or used topical therapeutic agents within the previous 48 h; had concomitant underlying skin disease (e.g., eczematous dermatitis), or were immunocompromised. First Nations’ patients were also excluded as treatment guidelines for these patients do not include the use of topical treatment regardless of severity, due to the high risk of developing post-streptococcal complications [20].

### 2.5. Randomisation

A local compounding pharmacist prepared the three treatment ointments in identical metal tubes. Treatment allocation was assigned by a computerised random sequence generator, such that patients, clinicians and investigators were blinded to the allocation, thereby minimising selection and confounding bias.

### 2.6. Intervention

The three treatment arms were: mupirocin, H_2_O_2_ and SWP. Ointments were applied 3 times a day for 5 days with a cotton bud and lesions covered with a standardised dressing between applications. Topical H_2_O_2_ is consistent with current New Zealand (NZ) [21,22] and United Kingdom (UK) [13] recommendations. Topical mupirocin is the first-line treatment for mild impetigo in Australian guidelines [9] and SWP is a vehicle in most ointments.

#### Procedure

At the initial visit (day 1), baseline data, including age, gender, occupation, smoking status, medications and relevant co-morbidities (e.g., diabetes and peripheral vascular disease), were collected using a standardised data collection form (File S1). The size, location and number of lesions were recorded on a body site map. Disposable rulers were placed in 2 axes near the lesions and a photograph was taken to allow objective assessment of changes in lesion parameters in post-study data analysis. A single wound swab was collected from each patient for microscopy, culture, and sensitivity (MCS) to characterise the local epidemiology and resistance patterns of bacteria, as well as guide any changes to treatment that may be required if the study treatment was not effective. For multiple skin lesions, the lesion swabbed was at the clinicians’ discretion.

Participants were provided with a standardised set of verbal and written instructions detailing the treatment protocol (File S2) and were advised to return for review at any time if they believed their condition had worsened. Treatment ointment (based on the randomisation schedule), cotton swabs and dressings were given to all patients. Participants were asked to complete a treatment diary (File S3) to encourage and measure treatment compliance. This also included a free-text feedback section for participants/guardians to share their thoughts on the treatment/study protocol. A standardised medical certificate was also provided if needed for work, school, or day-care. At the end of the initial visit, a follow-up appointment was scheduled for day 6 (post-treatment).

The follow-up visit on day 6 was a clinical review to determine if lesions had improved (reduced size and number) or if further treatment was required. Patients that had not improved were treated with oral antibiotics as per standard practice and swab MCS results were reviewed. Photographs of all lesions were taken as per the initial visit and any adverse reactions to treatment documented.

## 3. Results

### 3.1. Outcomes

**Figure 1:**
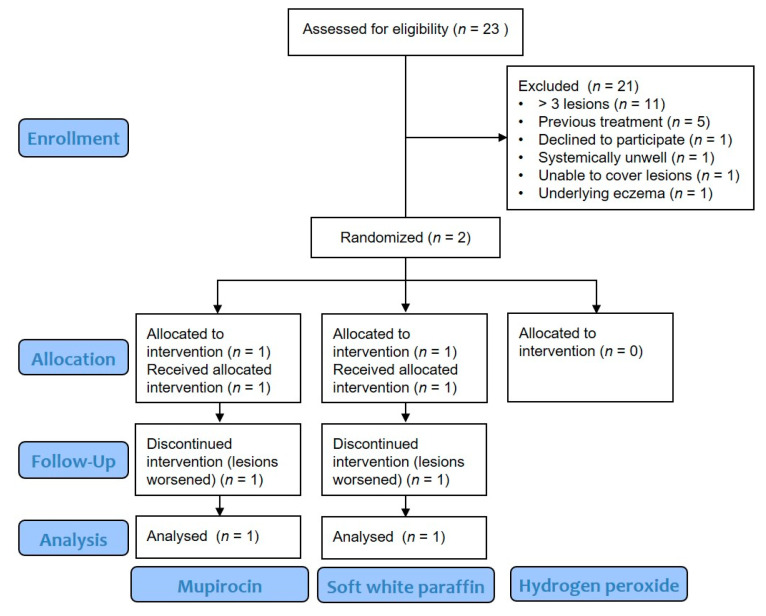
Consort flow diagram outlining treatment group pathways.

Twenty-three people were assessed for eligibility, of which 21 were excluded, most commonly due to having more than 3 lesions (*n* = 11) or previous treatment within the 48 h preceding presentation to the GP (*n* = 5) (Figure 1). Two children were recruited by two GPs, one at each of the participating practices.

One participant was allocated to the SWP arm and the other to the mupirocin arm of this study (Table 1). Both attended the follow-up visit (100% retention) and reported 100% adherence to the prescribed medication; however, only one patient/guardian completed the treatment diary. At clinical review, both patients were deemed to have worsened with larger and/or more lesions present and were subsequently prescribed oral antibiotics. One practice failed to measure or photograph the lesions at either timepoint due to time constraints in the practice and the additional protocol requirements were not deemed feasible. Neither patient reported adverse reactions to the treatment.

Ten GPs and one practice nurse were interviewed after recruitment concluded. Thematic analysis of their responses revealed several themes for recruitment barriers and improving trial delivery (Table 2), and a positive response to overall protocol acceptability.

#### 3.1.1. Barriers to Recruitment

Participating GPs believed they saw fewer impetigo presentations during the recruitment period than previous years. They hypothesized that social distancing, improved hand hygiene in children, hesitancy towards going out (including to visit the doctor) during the COVID-19 pandemic contributed to the reduction in impetigo presentations. GPs found that some patients presented after failed attempts with at-home topical treatment, including mupirocin ointment, phisohex wash and chlorsig ointment. A sub-theme that developed was how to escalate treatment after the use of expired topical treatments at home. Furthermore, by the time parents of patients present to the GP they have already been “putting up” (GP 4) with the impetigo lesions and want definitive therapy. Thus, being part of a trial is less appealing as they simply want this minor illness to pass so they can continue with work and school. GPs simply being busy and “having to remember” (GP 9) to recruit patients was another barrier to recruitment.

#### 3.1.2. Improved Delivery

Some GPs believed that, due to the rate of pre-treatment in patients presenting with impetigo, recruitment by other health practitioners (e.g., pharmacists and child health nurses) may be more effective. They also suggested that practices with more on-the-day bookings (e.g., large bulk-billing clinics), may have a higher rate of impetigo presentations and be more suited to a trial of this type. GPs in this study were established in the community with a minimum of two years practicing at their clinic, making them less likely to see acute conditions such as impetigo.

#### 3.1.3. Protocol Acceptability

Staff at one practice were happy with the study protocol, finding it “well thought out and logical” (GP 10), “easy to follow” (Practice Nurse) and “simple, unambiguous and pragmatic” (GP 10, PN). At the other practice, GPs were concerned the protocol added extra complexity to already busy GP consultations, for example, added time required to photograph and measure lesions.

## 4. Discussion

Impetigo is estimated to comprise 0.3% of GP consultations in Australia [23] and treatment impacts antimicrobial stewardship. Topical antiseptics present a potentially effective antibiotic-sparing treatment for mild cases. However, despite recommendations in several international guidelines, evidence supporting their use is poor. This trial aimed to determine the feasibility of an RCT that would determine the effectiveness of topical antiseptics compared to standard topical antibiotic treatment for impetigo to fill this gap in knowledge; however, we identified major barriers to recruitment.

### 4.1. Self-Initiated At-Home Treatments

Given the pragmatic nature of this study, many patients presenting to clinics with impetigo had already self-initiated treatments at home and thus were excluded. GPs discussed the high level of patient health literacy regarding impetigo and its treatment often as a result of sibling infections, or from advice on social media parenting groups. Many parents had superfluous mupirocin ointment from previous impetigo episodes and some GPs often had previously experienced opportunistic requests for mupirocin scripts at unrelated consultations to maintain a home supply.

An interesting sub-theme in the qualitative analysis was management of prior treatment with an expired topical antibiotic. This creates a conundrum for GPs as to whether the initial treatment has failed due to the product being expired or to it being truly ineffective for the presentation. One GP described a case of impetigo that had been treated with mupirocin ointment that was three years passed its expiration date. The GP–parent discussion focused on whether it was necessary to escalate treatment to oral antibiotics given lack of treatment efficacy could be due to use of mupirocin that had potentially lost its effectiveness over time. Better patient education about the use of expired prescriptions may be warranted, especially antibiotics given the global risk to health if stewardship is not considered. This education may best be delivered by community pharmacy.

To maintain methodological rigor, removal of “prior treatment” as an exclusion criterion was not possible. Guidelines for the prescription of topical antibiotics must be adhered to and strategies to connect with patients prior to home initiation of treatment should be considered.

### 4.2. Lesion Severity

The most common reason for exclusion from this study was too many lesions. A recent systematic review highlighted the lack of consensus on a definition of impetigo severity [11]. Descriptions for extensive impetigo ranged from general terms such as “widespread lesions” to more objective measures such as greater than 3 or 6 lesions or lesions affecting an area greater than 6 cm^2^ [11]. We opted for a conservative cutoff of lesion numbers (>3) given only topical treatments were being trialed. Based on the large number of patients excluded based on disease severity, GPs believed that other community practitioners who may see people at an earlier stage in the disease course would be better placed to recruit and deliver this protocol (e.g., community pharmacists and child health nurses). Other options could be partnering with local childcare centers in order to identify and refer impetigo cases to clinicians early. Careful selection of participating GP practices based on their demographics would be another strategy. While the practices included in this study had a young patient demographic, consideration must be given to waiting times for GP appointments. Practices that see more acute, on-the-day bookings, such as bulk-billing practices or practices with GP registrars [23,24], may be better suited to implementing this trial protocol. Future studies could be more inclusive with the threshold for the number of lesions allowed for recruitment, perhaps in line with the Médecins Sans Frontieres [25] and French [26] guidelines for the treatment of impetigo which indicate topical antibiotics for patients with less than 5 or 6 lesions, respectively.

## 5. Conclusions

Topical antiseptics present a potentially effective antibiotic-sparing treatment for mild impetigo. However, despite being recommended in several international guidelines, there is sparse evidence for topical antiseptic use. This trial aimed to determine the feasibility of an RCT that would determine the effectiveness of topical antiseptics compared to standard topical antibiotic treatment for impetigo to fill this gap in knowledge. The study protocol was found to be acceptable. Recruitment could be facilitated by targeting clinics with more on-the-day appointments and community pharmacists/child health nurses, thereby capturing participants with early stages of impetigo that have not been pre-treated with patient-initiated superfluous home antibiotics.

## Figures and Tables

**Table 1:** Patient demographics and clinical data at initial and follow-up visits.

	Patient 1	Patient 2
**Initial visit (Day 1)**		
Intervention	Mupirocin	Soft white paraffin
Age (years)	5	4
Gender	Male	Female
Occupation	Student	Student
Current medications	Nil	Nil
Co-morbidities	Nil	Nil
Number of lesions	3	3
Size of lesions	Not measured (photograph not taken)	1 mm × 1 mm
Wound swab	Not taken	No growth after 48 h
**Follow-up visit (Day 6)**		
Adherence	100% (reported verbally)	100%
Number of lesions	7	3
Size of lesions	Larger (not measured, no photograph taken)	2 mm × 2 mm
Adverse outcomes	Nil	Nil
Treatment diary	Not completed. Verbally conferred 100% adherence	Completed. 100% adherence
Outcome	Lesions worsened. Cephalexin commenced	Lesions ‘more crusty and slightly bigger’. Oral flucloxacillin commenced

**Table 2:** Themes resulting from content analysis of post-study interviews with practice staff.

	Theme	Selected Verbal Narrative
Barriers to Recruitment	Prior treatment	“parents have Bactroban from older siblings having impetigo and know what to do” (GP 2)
	GP workload, including impact of COVID-19	“Sometimes you just forget” (GP 6) “It’s hard to remember when you get busy” (GP 9)
	Impact of COVID-19 on infection control and impetigo incidence Impact of COVID-19 on GP attendence	“hand hygiene is being done with kids more” (GP 4) “most people have tried other things and just want definitive treatment” (GP 4)
Improving Trial Delivery	Involving other health practitioners	“child health nurses could see kids sooner” (GP 2) “Pharmacists might be better positioned to see people before the initial treatment” (GP 3)
	Practice demographics	“practices that aren’t booked out a week in advance” (GP 1)

GP = general practitioner.

## Data Availability

The data that support this study will be shared upon reasonable request to the corresponding author.

## Supplementary Materials

The following are available online at https://www.mdpi.com/article/10.3390/tropicalmed6040197/s1, File S1. Data collection form; File S2. Treatment protocol; File S3. Treatment diary.
