# Supplementary material for: Feasibility Study for a Randomised Controlled Trial for the Topical Treatment of Impetigo in Australian General Practice
Source: Trop Med Infect Dis. 2021 Nov 9;6(4):197. doi: 10.3390/tropicalmed6040197 (PMC8628881; doi:10.3390/tropicalmed6040197)
Supplement: Supplementary file 1 [file tropicalmed-06-00197-s001.zip › File S1.pdf]

Patient Record Number: _______

Registration form: To all participating medical practitioners and practice nurses, please kindly fill in this form for all consented patients with impetigo and those who do not meet the exclusion criteria:

1. <2 years
2. ≥4 skin lesions
3. Systemically unwell (e.g. fever, nausea, vomiting)
4. ATSI background
5. Lesions unable to be covered with dressing
6. Allergy to Mupirocin, hydrogen peroxide, soft white paraffin
7. Currently on antibiotics or using topical treatment in the last 48 hours
8. Underlying skin disease (e.g. eczema, scabies)
9. Immunocompromised
10. Significant language barrier

Date: ____________

Consent Form Completed Yes ☐ No ☐

Demographic

Patient Age (years): ______

Patient Gender: Female ☐ Male ☐ Other ☐

Ethnicity: ________________

Occupation: ______________

Smoker ☐ Non-smoker ☐

Co-morbidities (e.g. diabetes, PVD):

____

Current medications:

____

Patient Record Number: _______

Symptoms

1. Number of lesions: _______

2. Site of lesions

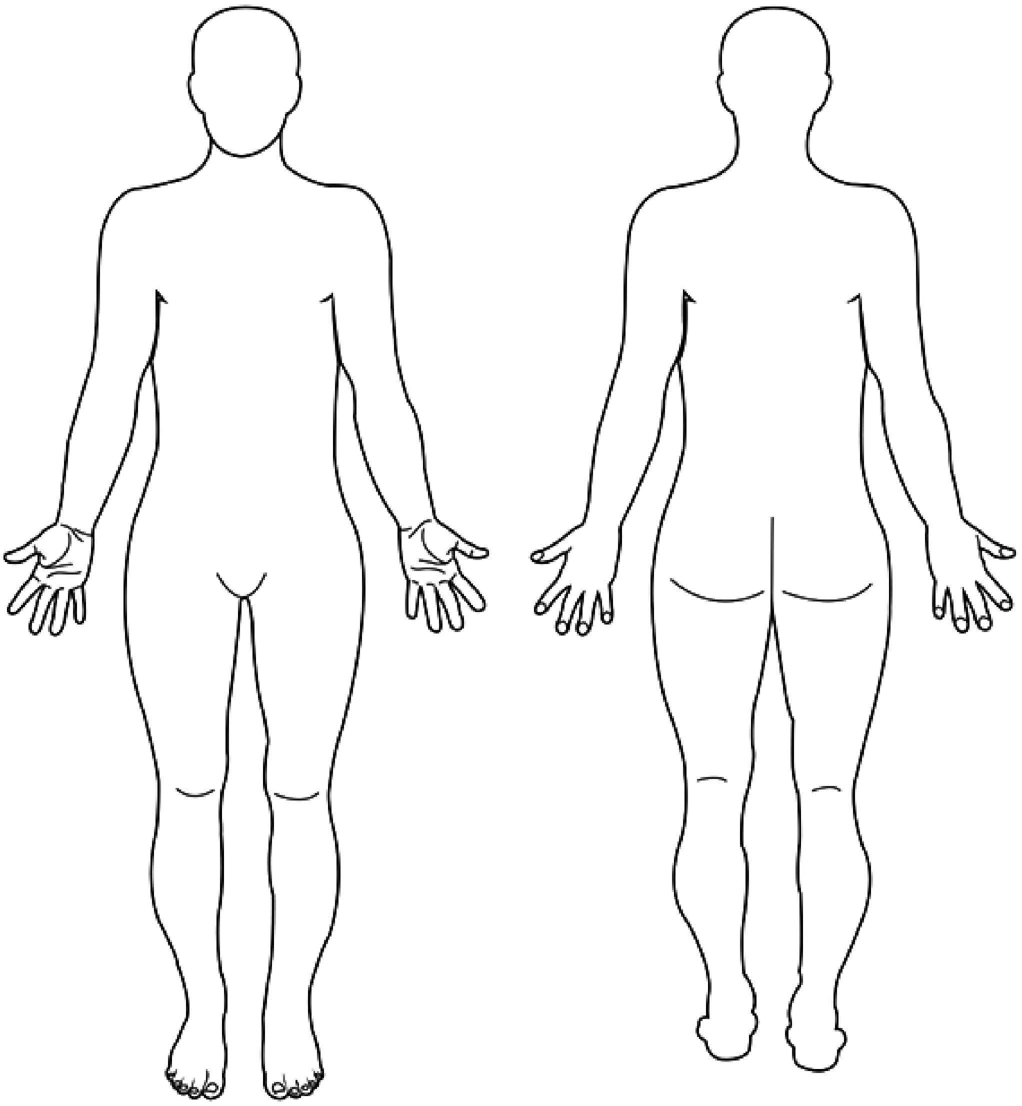

Treatment Allocation (envelope number): _______

Baseline Photo File #: ___________

**Wound Swab MCS taken:**	Yes	☐	No	☐
**Verbal Treatment Explanation:**	Yes	☐	No	☐
**Treatment Diary given:**	Yes	☐	No	☐

Patient Record Number: _______

Review Appointment

Date: ___________

Improved: ☐

Decrease in size of affected area or number of lesions or both such that no further treatment is required.

Not Improved: ☐

Further treatment required.

Details:

____

____

Reviewed early: ☐

Date

Reason (e.g. adverse reaction, not improved – fill in above):

____

____

Adverse reaction: ☐

Details:____

Review Photo File #:______________

Culture: ____

Sensitivities (resistance if any):

____

____

Treatment Diary returned: ☐
