# Supplementary material for: Feasibility Study for a Randomised Controlled Trial for the Topical Treatment of Impetigo in Australian General Practice
Source: Trop Med Infect Dis. 2021 Nov 9;6(4):197. doi: 10.3390/tropicalmed6040197 (PMC8628881; doi:10.3390/tropicalmed6040197)
Supplement: Supplementary file 1 [file tropicalmed-06-00197-s001.zip › File S2.pdf]

# Verbal Instructions to Participants

**Project:** Comparison of topical antibiotics with topical antiseptics and inert ointment for treatment of impetigo – protocol for a pilot prospective randomised double-blinded controlled trial for the topical treatment of impetigo in Australian general practice.

**Principal Investigator:** Dr Hilary Gorges
**Associate Investigators:** Professor Clare Heal, Dr Leanne Hall
**Institution:** James Cook University School of Medicine and Dentistry

1. Apply the ointment using a cotton bud to the skin lesion(s), three times a day for 5 days.

2. Cover the lesions with the dressings provided after each application.

3. The dressing can be left on overnight.

4. After you apply the ointment please check off the relevant box in the treatment diary [ ✓ ], if you miss an application please mark the relevant box [ X ]

5. (If child) You can return to day-care/school after 24 hours of treatment.

6. Return to see your GP on day 6/______ for a review appointment.

7. Return to see your GP sooner if you notice:
    a. Skin lesion(s) spreading or growing larger
    b. Redness or skin irritation around the area
    c. You become unwell
    d. You have any other queries or concerns.
